# Genomic Characterization of the Periwinkle Leaf Yellowing (PLY) Phytoplasmas in Taiwan

**DOI:** 10.3389/fmicb.2019.02194

**Published:** 2019-09-19

**Authors:** Shu-Ting Cho, Chan-Pin Lin, Chih-Horng Kuo

**Affiliations:** ^1^Institute of Plant and Microbial Biology, Academia Sinica, Taipei, Taiwan; ^2^Department of Plant Pathology and Microbiology, National Taiwan University, Taipei, Taiwan

**Keywords:** phytoplasma, plant pathogen, Mollicutes, genome, molecular evolution, effector

## Abstract

The periwinkle leaf yellowing (PLY) disease was first reported in Taiwan in 2005. This disease was caused by an uncultivated bacterium in the genus “*Candidatus* phytoplasma.” In subsequent years, this bacterium was linked to other plant diseases and caused losses in agriculture. For genomic investigation of this bacterium and its relatives, we conducted whole genome sequencing of a PLY phytoplasma from an infected periwinkle collected in Taoyuan. The *de novo* genome assembly produced eight contigs with a total length of 824,596 bp. The annotation contains 775 protein-coding genes, 63 pseudogenes, 32 tRNA genes, and two sets of rRNA operons. To characterize the genomic diversity across populations, a second strain that infects green onions in Yilan was collected for re-sequencing analysis. Comparison between these two strains identified 337 sequence polymorphisms and 10 structural variations. The metabolic pathway analysis indicated that the PLY phytoplasma genome contains two regions with highly conserved gene composition for carbohydrate metabolism. Intriguingly, each region contains several pseudogenes and the remaining functional genes in these two regions complement each other, suggesting a case of duplication followed by differential gene losses. Comparative analysis with other available phytoplasma genomes indicated that this PLY phytoplasma belongs to the 16SrI-B subgroup in the genus, with “*Candidatus* Phytoplasma asteris” that causes the onion yellowing (OY) disease in Japan as the closest known relative. For characterized effectors that these bacteria use to manipulate their plant hosts, the PLY phytoplasma has homologs for SAP11, SAP54/PHYL1, and TENGU. For genome structure comparison, we found that potential mobile unit (PMU) insertions may be the main factor that drives genome rearrangements in these bacteria. A total of 10 PMU-like regions were found in the PLY phytoplasma genome. Two of these PMUs were found to harbor one SAP11 homolog each, with one more similar to the 16SrI-B type and the other more similar to the 16SrI-A type, suggesting possible horizontal transfer. Taken together, this work provided a first look into population genomics of the PLY phytoplasmas in Taiwan, as well as identified several evolutionary processes that contributed to the genetic diversification of these plant-pathogenic bacteria.

## Introduction

Phytoplasmas are a group of plant-pathogenic bacteria with significant impact on agriculture worldwide (Lee et al., 2000; Hogenhout et al., 2008; Bertaccini and Lee, 2018). These wall-less bacteria belong to the class Mollicutes, with animal-pathogenic *Mycoplasma* and insect-symbiotic *Spiroplasma* as their close relatives (Gasparich, 2010; Chen et al., 2012). However, unlike *Mycoplasma* and *Spiroplasma*, axenic culture of phytoplasmas has not been successfully established yet, such that these bacteria are assigned to a “*Candidatus*” (“*Ca*.”) genus and lack formal taxonomy (The IRPCM Phytoplasma/Spiroplasma Working Team - Phytoplasma taxonomy group, 2004). For classification, the current system relies on the restriction fragment length polymorphism (RFLP) profiles of their 16S rRNA gene (Lee et al., 1993, 1998; Zhao et al., 2009; Zhao and Davis, 2016).

To date, >30 phytoplasmal 16S rRNA gene RFLP (16Sr) groups and many more subgroups have been established, with each group containing one or more “*Ca*. Phytoplasma” species (Zhao and Davis, 2016). Among these, “*Ca*. P. asteris” (Lee et al., 2004) that was assigned to the 16SrI group is the best characterized one. Strains belonging to “*Ca*. P. asteris” have a worldwide distribution and are associated with >100 economically important plant diseases. In Taiwan, a “*Ca*. P. asteris” phytoplasma that causes the periwinkle leaf yellowing (PLY) disease was first reported in Taoyuan in 2005 (Chen et al., 2011). In addition to leaf yellowing, this phytoplasma also induces other symptoms such as virescence, phyllody, and witches’ broom. Subsequent characterization of this phytoplasma revealed that its potential vectors include multiple leafhoppers, such as *Macrosteles orientalis*, *Cicadulina bipunctella*, *Phlogotettix cyclops*, and *Balclutha* sp. (Chen et al., 2011). Moreover, it can infect other cultivated plants such as chrysanthemum (*Chrysanthemum* sp.), cosmos (*Cosmos bipinnatus*), torenia (*Torenia fournieri*), Persian violet (*Exacum affine*), and cucumber (*Cucumis sativus*), also well as a weed, goosegrass (*Eleusine indica*) (Chen et al., 2011).

Since the initial disease report, this PLY phytoplasma continued to have recurring occurrences in Taoyuan and affected the commercial production of periwinkles. For more detailed characterization of this phytoplasma, we conducted whole genome sequencing of a PLY strain (i.e., strain DY2014; see section “Materials and Methods”) for comparative analysis with other 16SrI phytoplasmas. Particularly, we are interested in the gene content of this PLY phytoplasma with regard to effectors (Sugio et al., 2011b; Oshima et al., 2013). Moreover, to characterize the genomic diversity across different populations of the PLY phytoplasmas in Taiwan, a second strain (i.e., SS2016) was collected for re-sequencing analysis. The findings and implications are reported in this work.

## Materials and Methods

### Sample Collection and Characterization

Two strains of phytoplasmas were collected for this study. The first strain, DY2014, was collected from an infected periwinkle (*Catharanthus roseus*) in an herbal flower nursery located in the Dayuan District (Taoyuan City, Taiwan) in 2014. The second strain, SS2016, was collected from an infected green onion (*Allium fistulosum*) in a commercial field located in the Sanshing Township (Yilan County, Taiwan) in 2016. In both cases, the diseased plants were provided by the producers to the authors for diagnosis and research purpose.

After the initial symptom observation, total DNA of the infected plant was extracted using the Wizard Genomic DNA Purification Kit (Pomega, United States) for PCR-based detection of phytoplasmas and whole genome shotgun sequencing (Chung et al., 2013). For the periwinkle sample, leaf midribs were used for DNA extraction to increase the proportion of phytoplasma DNA. For the green onion sample, young leaves exhibiting the yellowing symptom were used without any attempt to cut out the vascular tissues. After the standard genomic DNA preparation, no additional enrichment for phytoplasma DNA (e.g., cesium chloride density-gradient centrifugation) was performed. The phytoplasma-specific primer set P1/P7 was used to amplify a partial sequence of the rRNA operon, followed by Sanger sequencing using an ABI Prism 3700 Genetic Analyzer (Applied Biosystems, United States). The resulting sequences were used as the queries to run BLASTN (Camacho et al., 2009) searches against the NCBI non-redundant nucleotide database (Benson et al., 2018) to verify the identity of the phytoplasma found. Furthermore, the 16Sr group assignment was performed by using iPhyClassifier (Zhao et al., 2009).

For the green onion sample, transmission electron microscopy (TEM) was performed to visualize phytoplasma cells within the phloem tissue. The sample was fixed with 2.5% glutaraldehyde and 4% paraformaldehyde in 0.1 M sodium phosphate buffer (pH 7.0) for 4 h at room temperature. Afterward, the sample was cleaned three times (20 min each) with the buffer and postfixed with 1% OsO_4_ in the same buffer for 4 h at room temperature, followed by three changes of the buffer (20 min each). The dehydration step was performed by using an ethanol series and propylene oxide. After dehydration, the sample was embedded in Spurr’s resin and sectioned with a Lecia Reichert Ultracut S ultramicrotome (Leica, Germany). The ultra-thin sections (70–90 nm) were stained with uranyl acetate and lead citrate. A FEI G2 Tecnai Spirit Twin transmission electron microscope (FEI, United States) at 80 KV was used for viewing and the images were taken with a Gatan Orius CCD camera (Gatan, United States).

### Genome Sequencing and Analysis

The procedures for genome sequencing and analysis were based on those described in our previous work on phytoplasma genomes (Chung et al., 2013; Orlovskis et al., 2017; Seruga Music et al., 2019). All bioinformatics tools were used with the default settings unless stated otherwise. For shotgun sequencing, one paired-end library with ∼550-bp insert size was prepared for each sample and sequenced using the Illumina MiSeq (Illumina, United States) platform.

The strain DY2014 was maintained by side grafting of periwinkles in a greenhouse at the National Taiwan University (Taipei, Taiwan). The genomic DNA used for whole genome shotgun sequencing was prepared from an artificially infected plant collected in 2016, 2 years after the initial collection of the naturally infected plant. The *de novo* assembly was performed using Velvet v1.2.10 (Zerbino and Birney, 2008) with the following settings: *k* = 191, exp_cov = auto, cov_cutoff = 10, max_coverage = 500, min_contig_lgth = 2000, and scaffolding = no. All contigs were used as the queries to run BLASTX (Camacho et al., 2009) searches against the NCBI non-redundant protein database (Benson et al., 2018) to identify putative phytoplasma contigs. The draft assembly was iteratively improved by mapping all raw reads to the contigs using BWA v0.7.17 (Li and Durbin, 2009) and checking for paired-reads that could extend the contigs, provide evidence for scaffolding, or fill gaps using SAMTOOLS v1.9 (Li et al., 2009) and IGV v2.5.0 (Robinson et al., 2011). Additionally, primer walking and Sanger sequencing were used to close the assembly gaps. The gene prediction was performed using RNAmmer v1.2 (Lagesen et al., 2007), tRNAscan-SE v1.3.1 (Lowe and Eddy, 1997), and Prodigal v2.6.3 (Hyatt et al., 2010). The homologous gene clusters among available phytoplasma genomes were identified by using OrthoMCL (Li et al., 2003). These results were used as the basis for annotation and comparative analysis. For manual curation of the annotation results, the KEGG database (Kanehisa et al., 2010) was used as the reference. Circos v0.69-6 (Krzywinski et al., 2009) was used for preparing the genome map. Mummer v3.23 (Kurtz et al., 2004) was used for inferring pairwise genome alignments with the setting “-maxmatch -c 2000.” For phylogenetic inference, MUSCLE v3.8.31 (Edgar, 2004) was used to generate multiple sequence alignments and PhyML v3.3 (Guindon and Gascuel, 2003) was used to infer maximum likelihood phylogenies.

For strain SS2016, the genomic DNA was prepared from a naturally infected green onion collected in 2016. A re-sequencing approach was used to identify its differences from DY2014. All Illumina raw reads derived from the SS2016 sample were mapped to the finalized assembly of DY2014 genome using BWA v0.7.17 (Li and Durbin, 2009). The mapping results were programmatically checked using the MPILEUP program in SAMTOOLS package v1.9 (Li et al., 2009), and visually inspected using IGV v2.5.0 (Robinson et al., 2011).

To predict the putative effector genes, all protein-coding genes were examined using SignalP v5.0 (Armenteros et al., 2019) to check for the presence of signal peptide based on the Gram-positive bacteria model. The program TMHMM v2.0 (Krogh et al., 2001) was used to verify that these putative secreted proteins have no transmembrane domain. Furthermore, the program COILS-WRAP (Lupas et al., 1991) was used to predict the presence of coiled coil domain with the settings “-m MTIDK -w 1”; regions with a probability of >50% in the sliding window analysis were identified. Finally, for those effectors that have been experimentally characterized (i.e., SAP11, SAP54/PHYL1, and TENGU), the functional domains involved in nuclear localization or virulence were identified based on the results of previous studies (Sugawara et al., 2013; Maejima et al., 2014; Sugio et al., 2014).

## Results and Discussion

### Symptom Observation and Phytoplasma Detection

**FIGURE 1:**
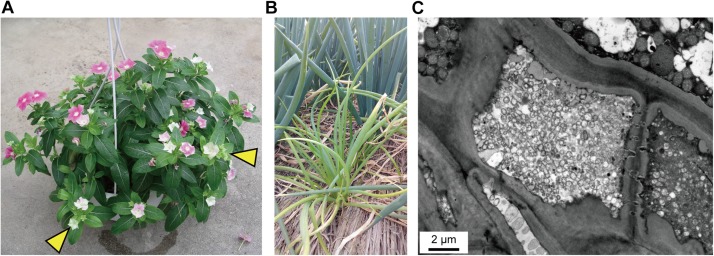
Disease symptoms. **(A)** Virescence (indicated by yellow arrowheads) on the periwinkle infected by strain DY2014. Photo provided by C-PL. **(B)** Chlorosis and dwarfism on green onion (front: infected by strain SS2016; back: healthy). Photo provided by Wei-An Tsai. **(C)** Transmission electron microscopy (TEM) observation of the sample in **(B)**; leaf longitudinal section. Note the large number of wall-less phytoplasma cells within the plant sieve element cell in the center.

The strain DY2014 is associated with the symptom of virescence on periwinkles (Figure 1A), as have been reported in the early characterization of PLY disease in the same region (i.e., Taoyuan, Taiwan) (Chen et al., 2011). The strain SS2016 is associated with chlorosis and dwarfism on green onions (Figure 1B). Furthermore, TEM observation of the leaf midvein confirmed that a large number of wall-less phytoplasma cells could be found within the plant sieve element cells (Figure 1C).

**FIGURE 2:**
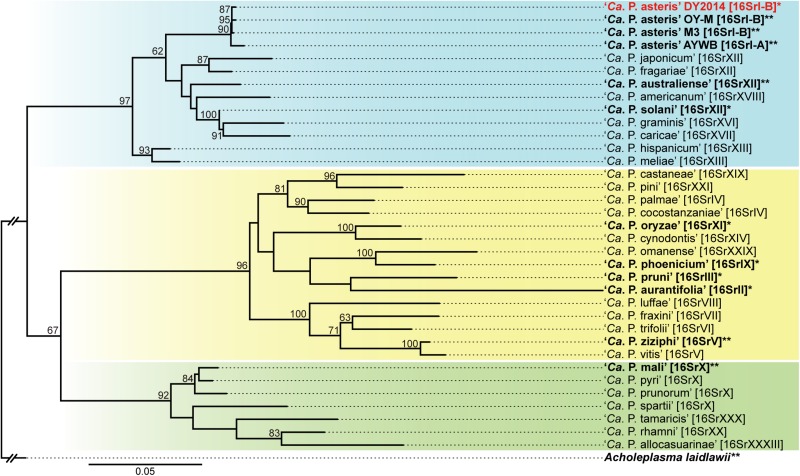
Maximum likelihood phylogeny of representative phytoplasmas based on the 16S rRNA gene. The numbers on the internal branches indicate the level of bootstrap support based on 1,000 resampling (only values ≥60% are shown). The strains with genome sequences available are highlighted in bold (^∗^draft; ^∗∗^complete). The 16Sr group assignments are provided in brackets. The three major clusters of phytoplasmas are highlighted by colored background (blue, yellow, and green). *Acholeplasma laidlawii* is included as the outgroup.

The molecular diagnostic based on 16S rDNA PCR found that both strains are 100% identical to the reference record of the PLY phytoplasma collected in 2005 from Taoyuan, Taiwan (GenBank accession FJ437568). Based on the *in silico* RFLP analysis by iPhyClassifier (Zhao et al., 2009), this sequence may represent a new subgroup within the 16SrI group and is most similar to the 16SrI-B representative (GenBank accession AP006628) with a similarity coefficient of 0.97. The maximum likelihood phylogeny based on the 16S rRNA gene provided a similar inference, namely that these PLY phytoplasma strains in Taiwan are most closely related to the 16SrI-B onion yellows (OY-M) phytoplasma found in Japan (Figure 2).

### Genomic Characterization

**FIGURE 3:**
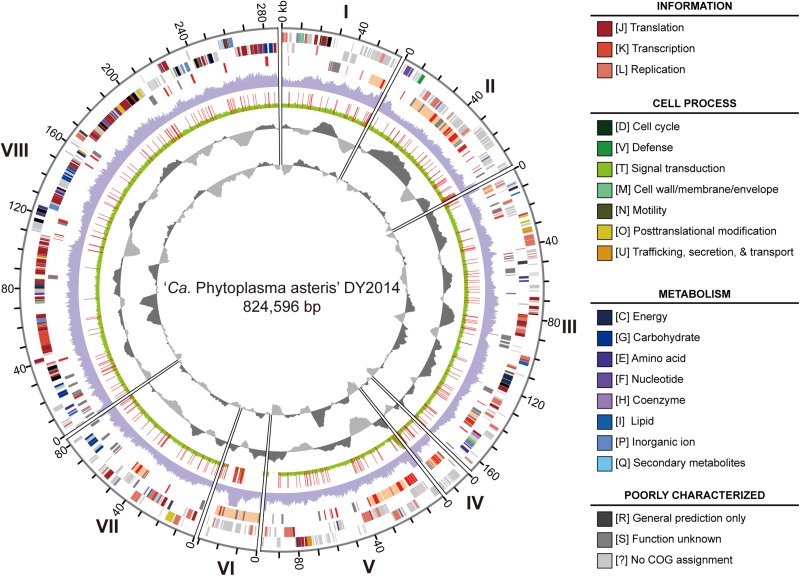
Genome map of the periwinkle yellowing phytoplasma DY2014. Rings from the outside in: (1) contig id (I-VIII); (2) scale marks (kb); (3 and 4) protein-coding genes on the forward and reverse strand, respectively (color-coded by functional category); (5) PMU-like regions (orange), putative secreted proteins (red), and pseudogenes (gray); (6) sequencing coverage of DY2014 (purple); (7) sequencing coverage of SS2016 (green), polymorphic sites in comparison with DY2014 are highlighted in red; (8) GC skew (dark gray: positive; light gray: negative); (9) GC content (dark gray: above average; light gray: below average).

The genome sequencing of these two phytoplasma strains generated ∼5.2 Gb of raw reads for DY2014 (NCBI accession: PRJNA530090) and ∼4.6 Gb for SS2016 (PRJNA529747). For DY2014 that was found in an infected periwinkle, ∼1.9% of the Illumina reads were mapped to the finalized phytoplasma assembly, while the remaining reads were mostly derived from the plant host. This sequencing depth corresponded to ∼124-fold coverage of the phytoplasma genome and the *de novo* assembly produced eight contigs totaling 824,596 bp (Figure 3). These contigs can be arranged into one linear scaffold based on PCR confirmation; only the connection between contigs II and III was not verified. Unfortunately, due to the high sequence composition biases (i.e., 27.6% genome-wide G + C content) and highly abundant repeats (e.g., homopolymers and di-/tri-nucleotide repeats), the Sanger sequencing of those PCR products were unsuccessful and we were unable to close those assembly gaps.

For strain SS2016 that was derived from an infected green onion, only ∼0.17% of the Illumina reads were assigned to phytoplasma, which corresponded to ∼11-fold coverage of this bacterial genome. *De novo* assembly with such low coverage was not feasible so a re-sequencing analysis was performed by using the DY2014 genome as the reference. Based on the mapping results, 10 structural variations and 337 sequence polymorphisms were found between these two genomes (Figure 3 and Supplementary Table S1). Among these sequence polymorphisms, 201 (60%) were found within protein-coding regions. These include six indels that affected six different genes (two encode putative secreted proteins and four encode hypothetical proteins) and 123 single nucleotide polymorphisms (SNPs) that introduced non-synonymous mutations in 93 protein-coding genes. Compared to a previous population genomics study of the maize bushy stunt phytoplasmas (MBSP) collected in Brazil (Orlovskis et al., 2017), the genetic divergence observed between these two PLY phytoplasmas in Taiwan is much higher. For the MBSP study, six strains were collected from two locations in the state of São Paulo (i.e., Guaíra and Piracicaba; ∼280 km apart) and only 86 sequence polymorphisms were found. In comparison, the two PLY strains included in this study were collected from locations that are ∼63 km apart. Even after adjusting for the difference in genome size, the density of polymorphic sites in these PLY phytoplasmas is still >twofold higher. Because those six MBSP strains were all collected from infected maize plants, while the two PLY strains in this study were collected from different plant hosts (i.e., periwinkle and green onion), it is possible that the specialization for different hosts may have contributed to the genetic divergence observed. Nevertheless, many other factors may be involved in shaping the genetic diversity of phytoplasmas. For example, although Taoyuan and Yilan have nearly the same average monthly temperatures, Yilan is located in the eastern coast of Taiwan and receives much more rainfall during winter. Additionally, compared to the large maize farms in Brazil, the periwinkle and green onion production in Taiwan are operated at much smaller scales, which may restrict the effective population sizes of phytoplasmas and their vectors/hosts, thus resulting in higher levels of genetic drift. Given these complexities, it is possible that no straightforward approach exists for quantifying the relative contribution of each factor to phytoplasma genetic diversity.

Despite the polymorphisms found, the genome-wide sequence identity between these two PLY phytoplasmas is >99.9%. Base on this high similarity, as well as the availability of a high-quality *de novo* assembly, DY2014 was selected as the representative for more detailed analysis and comparison with other phytoplasmas. The annotation of this DY2014 draft assembly (SRMC00000000.1) contains two complete sets of 16S-23S-5S rRNA gene clusters, 32 tRNA genes covering all 20 amino acids, 775 protein-coding genes, and 63 pseudogenes (Table 1). Similar to other phytoplasmas with complete genome sequences available, particularly those belonging to “*Ca*. P. asteris” within the 16SrI group (Oshima et al., 2004; Bai et al., 2006; Orlovskis et al., 2017), the gene content and metabolic pathways are highly reduced. Among the protein-coding genes, 264 (34%) were annotated as hypothetical proteins and 424 (55%) lacked any specific COG functional category assignment. For those with functional category assignments, 206 (27% of total, 59% among those with COG assignments) are related to the processing of genetic information (i.e., translation [J], transcription [K], and replication [L]). Other than information processing, genes encode putative secreted proteins (76; 10% of total) and transporters (44; 6% of total) are two of the largest groups. These findings are similar to the results from previous characterization of phytoplasma genomes (Kube et al., 2012; Chung et al., 2013; Oshima et al., 2013; Chen et al., 2014; Seruga Music et al., 2019). Two major inferences could be made from these observations. First, a substantial fraction of phytoplasma genes are still yet to be characterized and the current reference databases are inadequate. Second, the overall gene content of all characterized phytoplasma genomes fits the profile of obligate parasites that rely on hosts for survival and replication. Other than the genes necessary for processing their own genetic information, substantial fractions of genes are devoted to manipulate their hosts (e.g., secreted proteins) and to acquire nutrients (e.g., transporters).

**TABLE 1:** Genome statistics of representative “*Ca*. P. asteris” strains.

**Strain**	**DY2014**	**OY-M**	**M3**	**AYWB**
16Sr group assignment	16SrI-B	16SrI-B	16SrI-B	16SrI-A
Host	Periwinkle (*Catharanthus roseus*)	Onion (*Allium cepa*)	Maize (*Zea mays*)	Lettuce (*Lactuca sativa*)
Source	Taiwan	Japan	Brazil	United States
Assembly status	Draft, eight contigs	Complete	Complete	Complete
GenBank accession	SRMC01000001–SRMC01000008	NC_005303	NZ_CP015149	NC_007716
Genome size (bp)	824,596	853,092	576,118	706,569
G + C content (%)	27.6	27.8	28.5	26.9
Coding density (%)	70.6	72.8	75.1	73.5
Number of rRNA genes	6	6	6	6
Number of tRNA genes	32	32	32	32
Number of protein-coding genes	775	728	498	557
Number of pseudogenes	63	144	34	116

**FIGURE 4:**
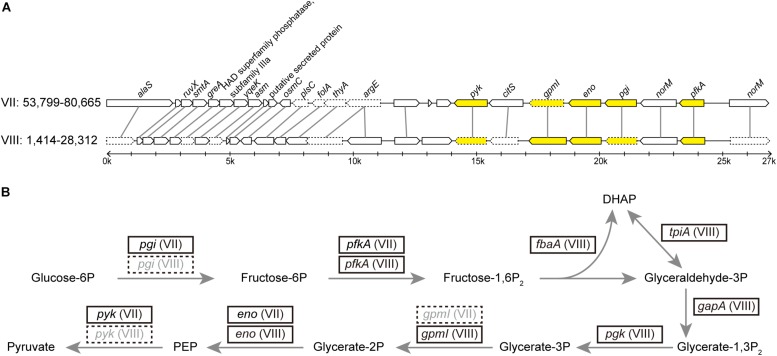
**(A)** Synteny between two duplicated regions within the DY2014 genome. The contig ids (i.e., VII and VIII) and the start-end positions of these regions are labeled. Genes participated in the glycolysis are highlighted in yellow. Intact genes are drawn with solid borders, putative pseudogenes are drawn with dotted borders. **(B)** Glycolysis pathway and the genes involved. Four of these genes (i.e., *fbaA*, *tpiA*, *gapA*, and *pgk*) are located in other regions of contig VIII. Correspondence between gene name and product: *pgi*, glucose-6-phosphate isomerase; *pfkA*, 6-phosphofructokinase; *fbaA*, fructose-bisphosphate aldolase; *tpiA*, triosephosphate isomerase; *gapA*, glyceraldehyde-3-phosphate dehydrogenase; *pgk*, 3-phosphoglycerate kinase; *gpmI*, phosphoglycerate mutase; *eno*, enolase; *pyk*, pyruvate kinase.

Compare to other bacteria that form obligate symbiosis with their hosts, phytoplasmas are similar in having small genomes but are distinct in having high levels of genomic plasticity (McCutcheon and Moran, 2012). We found a set of two 27-kb duplicated regions in this PLY phytoplasma (Figure 4). These regions contain genes involved in glycolysis pathway, which is the major energy-yielding pathway for phytoplasmas (Oshima et al., 2004, 2013; Kube et al., 2012). Intriguingly, differential gene losses have occurred in these two regions, such that both regions are required for the complete glycolysis pathway. Based on a model proposed for symbiont genome evolution (Lo et al., 2016), it is expected that eventually those pseudogenes will be removed through accumulation of mutations due to the deletional biases observed in bacterial genomes (Mira et al., 2001; Kuo and Ochman, 2009). At that point, the disruption of synteny may appear to have been resulted from multiple events of translocation that involved few genes at a time.

A similar finding regarding the duplication of glycolytic genes has been reported in an onion yellows phytoplasma strain OY-W (Oshima et al., 2007). Intriguingly, OY-W causes severe symptoms in its plant hosts, while its closely related strain OY-M causes only mild symptoms of leaf yellowing and has only one glycolytic gene cluster, suggesting that gene duplications may affect bacterial physiology and their virulence.

### Comparative Genomics Among 16SrI Group Phytoplasmas

**FIGURE 5:**
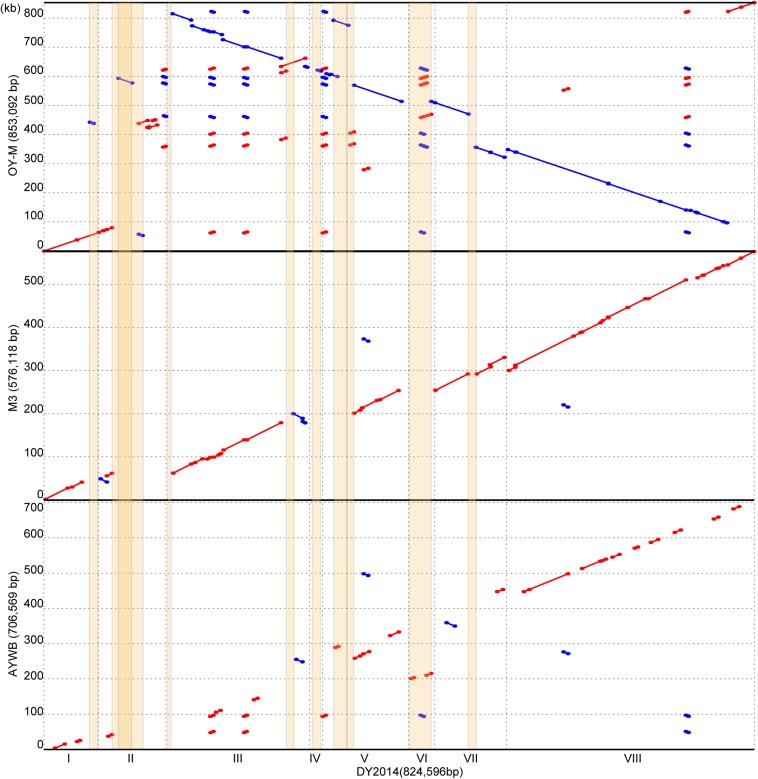
Pairwise genome alignments. The genome of DY2014 was used as the reference for comparison with other 16SrI group representatives. Matches on the same strand and the opposite strand are labeled in red and blue, respectively. PMU regions are highlighted in orange. A region on contig II has a darker shade of orange due to the insertion of PMU2 into the middle of PMU3 (see Supplementary Figure S1).

In phytoplasma genome organization and evolution, putative mobile units (PMUs) are known to play an important role (Bai et al., 2006). A total of 10 PMUs were found in this DY2014 genome (Supplementary Figure S1), including a set of nested PMUs (i.e., PMU2 was inserted within PMU3, disrupting the *dnaB* of the latter). Based on pairwise genome alignments, most of the synteny breakpoints correspond to PMU insertion sites (Figure 5). Although DY2014 is most closely related to OY-M based on 16S phylogeny (Figure 2), genome characteristics, and geographical locations (Table 1), the overall chromosome structure differ considerably between these two strains (Figure 5). This lack of conservation appeared to be resulted from a large inversion and multiple expansions of repetitive regions in the OY-M genome. If the PMUs were excluded, DY2014 has a chromosomal organization that is highly similar to the MBSP strain M3, which is a 16SrI-B strain with a much smaller genome (Table 1). Such conservation could be found even in the 16SrI-A strain AYWB (Figure 5). Based on these observations, it is likely that the increase of PMU copy numbers and genome sizes observed in DY2014 and OY-M represent derived traits. Nevertheless, more high-quality genome assemblies are necessary for examining the contribution of PMU abundance to phytoplasma genome size variations in general.

**FIGURE 6:**
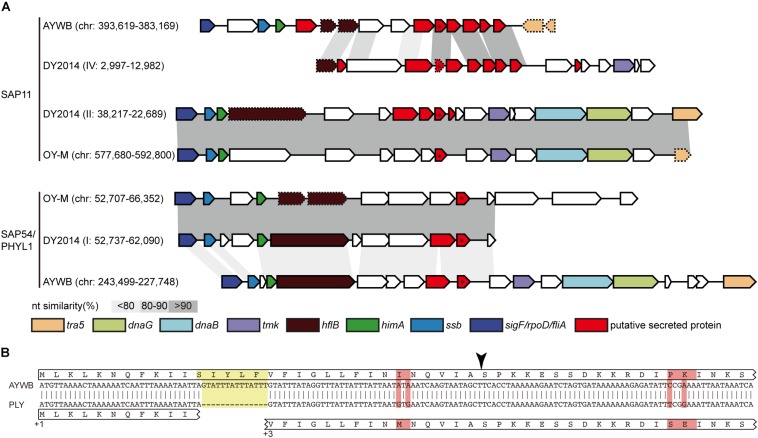
**(A)** Comparison of effector-containing putative mobile units (PMUs). The genomic location (contig: start-end) of each PMU is labeled (“chr” is the abbreviation of chromosome for strains with complete genome sequence). Genes that are commonly associated with PMUs are highlighted in colors, putative secreted proteins are colored in red. The two focal effector genes, SAP11 (top) and SAP54/PHYL1 (bottom), are labeled with an asterisk. Intact genes are drawn with solid borders, putative pseudogenes are drawn with dotted borders. Nucleotide sequence similarities between conserved regions are illustrated by different shades of gray colors. **(B)** Sequence alignment to illustrate a 14-bp deletion (highlighted in yellow) in the contig IV of DY2014 that resulted in a frameshift mutation in its SAP11 homolog. The reading frames (i.e., +1 and +3) are labeled. Polymorphic sites are highlighted in red. The black arrowhead indicated the putative signal peptide cleavage site.

In addition to genome size expansion and chromosomal restructuring, PMUs also play an important role in phytoplasma evolution because these mobile genetic elements often carry putative effector genes that these bacteria use to manipulate their hosts (Bai et al., 2006; Sugio and Hogenhout, 2012). Horizontal transfer of PMUs between diverse phytoplasmas have been inferred for several lineages within the genus (Chung et al., 2013; Ku et al., 2013; Seruga Music et al., 2019), suggesting that effector gene exchanges were prevalent in their evolutionary history. Moreover, sequence polymorphisms for a putative effector gene located on a PMU among field isolates of the MBSP in Brazil have been shown to be correlated with the symptoms induced by these bacteria (Orlovskis et al., 2017). Examination of the three phytoplasma effectors that have been functionally characterized, namely SAP11 (Bai et al., 2009; Sugio et al., 2011a, 2014; Lu et al., 2014; Chang et al., 2018), SAP54/PHYL1 (MacLean et al., 2011, 2014; Maejima et al., 2014; Orlovskis and Hogenhout, 2016), and TENGU (Hoshi et al., 2009; Sugawara et al., 2013; Minato et al., 2014), found that the homologous genes for all these effectors are present in these two PLY phytoplasma strains in Taiwan. Comparison of these effector genes between the two PLY phytoplasmas found only one non-synonymous mutation affecting a SAP11 homolog (locus tag: PLY_1110). This low level of sequence divergence may be explained by purifying selection acting on these important genes, or the lack of sufficient divergence time for mutation accumulation. Similar to other 16SrI phytoplasmas, the SAP11 and SAP54/PHYL1 homologs in PLY phytoplasma are all located within PMUs (Figure 6).

**FIGURE 7:**
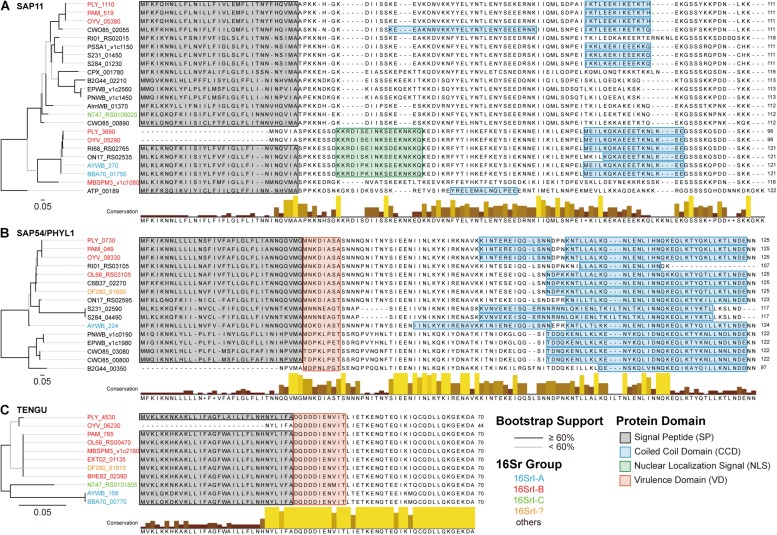
Comparison of phytoplasma effectors. **(A)** SAP11 homologs, **(B)** SAP54/PHYL1 homologs, and **(C)** TENGU homologs. Left, maximum likelihood phylogenies based on the protein sequence alignments. Branches colored in black indicate those with a bootstrap support of ≥60%, those in gray have a bootstrap support of <60%. The locus tags are color-coded according to the 16Sr group assignments. Right, multiple sequence alignments with highlights for functional domains.

Intriguingly, these two PLY phytoplasma strains in Taiwan (i.e., DY2014 and SS2016), as well as one onion yellows strain in Japan (i.e., OY-V) (Kakizawa et al., 2014), were found to harbor two copies of SAP11 gene. In addition to a vertically inherited 16SrI-B type copy (locus tag: PLY_1110), a second copy may have originated from horizontal acquisition of a 16SrI-A type PMU that exhibited synteny conservation with one PMU-like region in the AYWB phytoplasma genome (Figure 6A). Other than synteny conservation in the neighboring regions, multiple sequence alignment and molecular phylogenetic analysis further support that these PLY/OY-V SAP11 homologs could be classified into two types (Figure 7). Based on the previous characterization of 16SrI group phytoplasmas (Lee et al., 2004), 16SrI-A strains are mainly distributed in the eastern United States and infect Asteraceae hosts, while 16SrI-B strains are found in the western United States as well as worldwide and have highly diverse plant hosts (e.g., those infect celery in California, maize in Brazil, onion in Japan, periwinkle in Taiwan, etc). Thus, it is plausible that the United States is the location for these phytoplasmas to have gene flow. More comprehensive sampling of phytoplasma genomes, particularly those 16SrI-B strains in the United States, is necessary to test such hypothesis.

Regarding the putatively acquired 16SrI-A type SAP11 in PLY (locus tag: PLY_3690), a 14-bp deletion in the coding region has introduced a frameshift mutation (Figure 6B). Although an alternative start codon may be used for translation, this truncated product lacks the full-length signal peptide conserved in other SAP11 homologs (Figure 7A) and may not be functional. A previous work that characterized the function of several SAP11 homologs has demonstrated that the AYWB SAP11 (i.e., 16SrI-A type) exhibited strong localization to plant cell nuclei and induced severe phenotypes of leaf crinkling in *Arabidopsis thaliana*, while the OY-M homolog (i.e., 16SrI-B type) did not (Chang et al., 2018). It is unclear if there is any advantage for a phytoplasma to harbor both types of SAP11 homologs.

The presence and localization of coiled coil domains exhibited some variations among SAP11 homologs (Figure 7A). For example, in the central part of sequences, homologs belonging to the 16SrI-B type (e.g., PLY_1110, PAM_519, and OYV_05380) all exhibited a weak signal (i.e., ∼10% probability) for an additional coiled coil domain, yet CWO85_02055 (from “*Candidatus* Phytoplasma ziziphi” in the 16SrV group) is the only one that reached the significance threshold (i.e., 60–70% probability). Similar to these findings for SAP11 homologs, SAP54/PHYL1 homologs also exhibited high variation in the exact boundaries of their coiled coil domains despite having high sequence similarities (Figure 7B). These findings require future experimental studies to validate the bioinformatic inference and to establish the biological significance. Finally, in contrast to the nearly genus-wide distribution of SAP11 and SAP54/PHYL1 homologs, TENGU homologs appeared to be restricted to those 16SrI group phytoplasmas and have extremely high levels of sequence conservation (Figure 7C).

## Conclusion

The results from this work provided insights into the genomic diversity among phytoplasmas at the levels of populations and species. When comparing across populations, differentiations in plant hosts may play a more important role than geographical distance in shaping genetic diversification. However, such inference requires more comprehensive sampling of closely related phytoplasma genomes for further tests. At the level of species or sub-species comparisons, mobile genetic elements such as PMUs played an important role in genome rearrangement, gene content evolution, and horizontal gene transfer. Particularly, the exchange of effector genes among different phytoplasma lineages may facilitate their adaptation. Furthermore, duplication of chromosomal segments followed by differential gene loss, such as that commonly observed after whole genome duplication in eukaryotes (Kellis et al., 2004), could be another evolutionary process the drives the genome divergence of these bacteria. This finding provided novel insights into the genome evolution of pathogenic bacteria with highly reduced genomes.

## Data Availability Statement

The Illumina raw reads were deposited in NCBI Sequence Read Archive (SRA) under the accessions PRJNA530090 (strain DY2014) and PRJNA529747 (strain SS2016). The *de novo* genome assembly of DY2014 was deposited in GenBank under the accession SRMC00000000. The version described in this manuscript is version SRMC01000000. The genomic variations between these two strains are provided in the Supplementary Material.

## Author Contributions

C-PL provided the biological materials. S-TC, C-PL, and C-HK performed the experiments. S-TC and C-HK analyzed the data. S-TC prepared the figures and Supplementary Material. C-HK designed the experiments, acquired the funding, wrote the manuscript, and supervised the project.

## Conflict of Interest

The authors declare that the research was conducted in the absence of any commercial or financial relationships that could be construed as a potential conflict of interest.

## Supplementary Material

FIGURE S1 Organization of putative mobile units (PMUs) in the DY2014 genome. The genomic location of each PMU (i.e., contig id and start-end position) is provided. Note that PMU2 is located within PMU3.

TABLE S1 Polymorphic sites between the two phytoplasma strains characterized in this study.
