# Supplementary figures and images for: Genomic Characterization of the Periwinkle Leaf Yellowing (PLY) Phytoplasmas in Taiwan
Source: Front Microbiol. 2019 Sep 19;10:2194. doi: 10.3389/fmicb.2019.02194 (PMC6761752; doi:10.3389/fmicb.2019.02194)

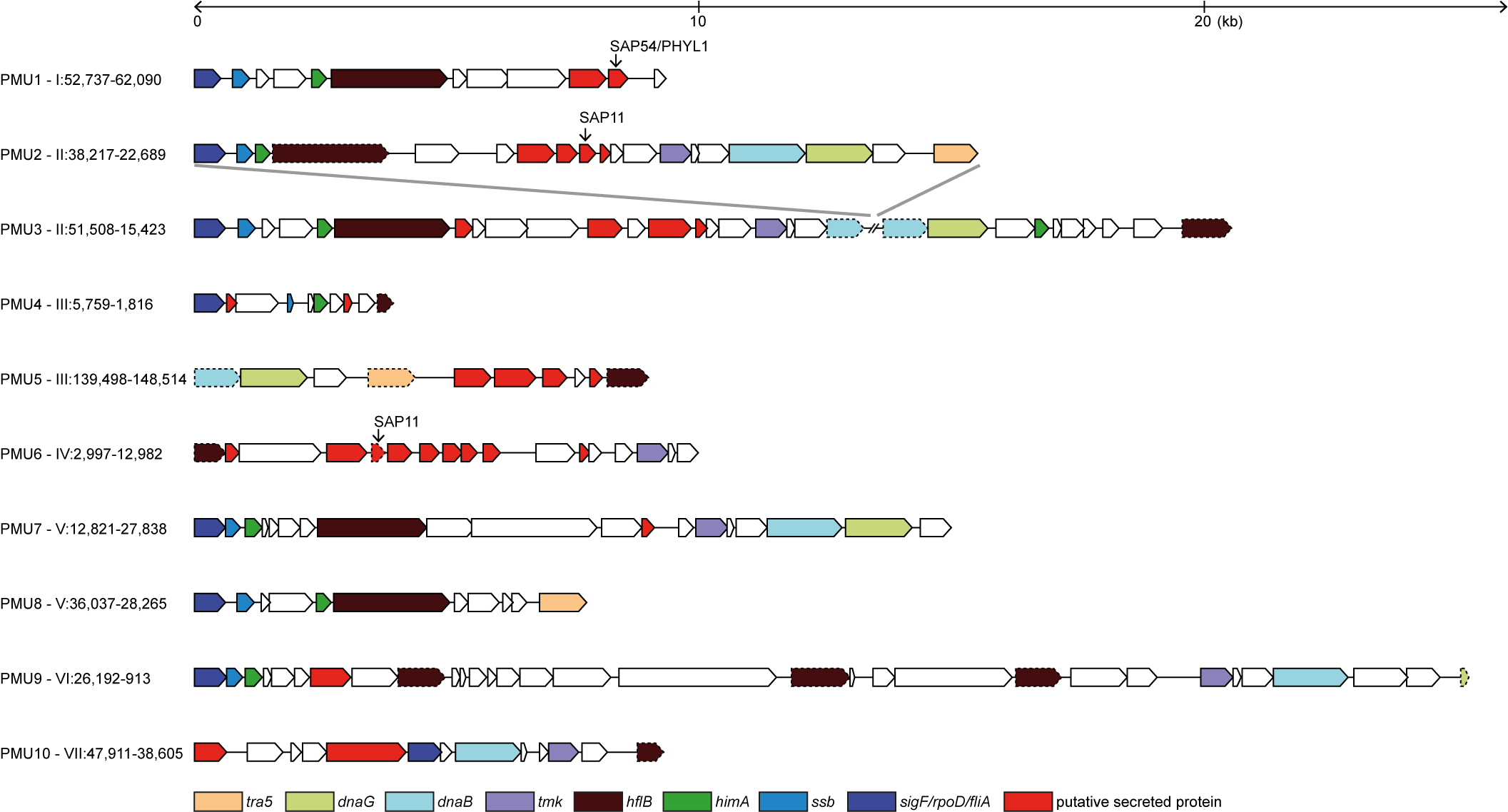

Supplement: FIGURE S1 — Organization of putative mobile units (PMUs) in the DY2014 genome. The genomic location of each PMU (i.e., contig id and start-end position) is provided. Note that PMU2 is located within PMU3. [file Image_1.TIF]
